# Spirometry in Healthy Subjects: Do Technical Details of the Test Procedure Affect the Results?

**DOI:** 10.1371/journal.pone.0107782

**Published:** 2014-09-22

**Authors:** Luciana Sipoli, Larissa Martinez, Leila Donária, Vanessa Suziane Probst, Graciane Laender Moreira, Fabio Pitta

**Affiliations:** 1 Laboratory of Research in Respiratory Physiotherapy (LFIP), Departament of Physiotherapy, Universidade Estadual de Londrina (UEL), Londrina, Paraná, Brazil; 2 Research Center in Health Sciences, Universidade Norte do Paraná (UNOPAR), Londrina, Paraná, Brazil; University of Münster, Germany

## Abstract

**Introduction:**

Spirometry should follow strict quality criteria. The American Thoracic Society (ATS) recommends the use of a noseclip; however there are controversies about its need. ATS also indicates that tests should be done in the sitting position, but there are no recommendations neither about position of the upper limbs and lower limbs nor about who should hold the mouthpiece while performing the maneuvers: evaluated subject or evaluator.

**Objectives:**

To compare noseclip use or not, different upper and lower limbs positions and who holds the mouthpiece, verifying if these technical details affect spirometric results in healthy adults.

**Methods:**

One hundred and three healthy individuals (41 men; age: 47 [33–58] years; normal lung function: FEV_1_/FVC = 83±5, FEV_1_ = 94 [88–104]%predicted, FVC = 92 [84–102]%predicted) underwent a protocol consisting of four spirometric comparative analysis in the sitting position: 1) maximum voluntary ventilation (MVV) with vs without noseclip; 2) FVC performed with *vs* without upper limbs support; 3) FVC performed with lower limbs crossed *vs* lower limbs in neutral position; 4) FVC, slow vital capacity and MVV comparing the evaluated subject holding the mouthpiece *vs* evaluator holding it.

**Results:**

Different spirometric variables presented statistically significant difference (p<0.05) when analysing the four comparisons; however, none of them showed any variation larger than those considered as acceptable according to the ATS reproducibility criteria.

**Conclusions:**

There was no relevant variation in spirometric results when analyzing technical details such as noseclip use during MVV, upper and lower limb positions and who holds the mouthpiece when performing the tests in healthy adults.

## Introduction

Spirometry is a respiratory assessment which allows diagnosis and quantification of lung diseases [1]. This assessment should follow strict quality control criteria based on international guidelines such as those published by the American Thoracic Society (ATS) and European Respiratory Society (ERS) [2].

One of the common recommendations for the spirometric test is the use of a noseclip [1], [2]. However, this is in contrast to the scientific literature, since a reasonable body of literature which analyzed the noseclip's use in maneuvers of forced vital capacity (FVC) and slow vital capacity (VC) did not find differences in the spirometric results when using or not the device [3]–[5]. Concerning the maximum voluntary ventilation (MVV), however, only two studies analyzed the use of a noseclip, one involving patients with chronic obstructive pulmonary disease (COPD) [6] and the other young healthy adults [7], and in both there was no statistically significant difference when comparing its use or not. In addition, these two studies also analyzed subjective discomfort with the noseclip [6], [7].

Regarding the body posture recommended by the ATS/ERS guidelines, the tests should be preferably done in the sitting position, but if the standing or lying position is used, the evaluator should report it [2] since it is known that the variation between lying, sitting and standing interferes with the test results [1]. Furthermore, it is mentioned that cervical flexion causes a decrease in peak expiratory flow (PEF) [2], whereas the II Brazilian Consensus of Spirometry, a Brazilian document concerning the recommendations for spirometry testing, specifies that the neck should be maintained in a neutral position [1] because its flexion and extension decreases and increases forced expiratory volume in one second and forced vital capacity ratio (FEV_1_/FVC) and FVC, respectively [3]. However, neither the ATS/ERS guidelines nor any other scientific guideline bring any recommendation about the positioning of upper limbs (UL) (i.e., with or without support on the arm chair), and lower limbs (LL) (i.e., flexed or extended knee, with or without hip adduction). In addition, it is not specified who should hold the mouthpiece, evaluator or evaluated subject. In clinical practice, there are centers where the evaluator holds the mouthpiece, and there are other centers where the subjects are instructed to hold the mouthpiece themselves [2].

Since it is not known whether technical details of the spirometric test interfere with the accuracy of its results, it is necessary to investigate these issues in order to contribute to clinical practice and future scientific studies.

The objectives of this study were: 1) to assess whether the use of a noseclip interferes with MVV testing results in healthy individuals; 2) to evaluate discomfort during MVV performed with and without a noseclip in healthy individuals; 3) to analyze whether changes in the positioning of upper and lower limbs interfere with spirometric results in healthy subjects when tests are performed in the sitting position, 4) to verify if there is a difference in spirometric results when the evaluator holds the mouthpiece compared to the evaluated subject holding it.

## Methods

### Design, inclusion and exclusion criteria

This was a cross-sectional, observational study with a convenience sample, conducted at the Laboratory of Research in Respiratory Phisiotherapy (LFIP) located at the University Hospital of the Universidade Estadual de Londrina (HU-UEL), Brazil.

Participants were recruited from the community (January to July 2013) through informal announcements, and were mostly relatives of University students and employees (or employees' family members) of a University Hospital.

Participants met the following inclusion criteria: 1) individuals of both genders aged 18 years or more; 2) absence of conditions which can alter lung or chest wall compliance, such as kyphoscoliosis and neuromuscular disease; 3) absence of hemoptysis or recent angina, retinal detachment, arterial hypertensive crisis, pulmonary edema and thoracic aorta aneurysm. Participants who had spirometric values outside the normal range [9], those who did not understand/comply with the tests and those who had no physical conditions to perform the proposed procedures would be excluded.

### Ethics Statement

The study was approved by the Ethics Committee of UEL (Number 074/2012), and all participants gave written informed consent according to the guidelines of UEL ethical review board prior to their inclusion in the present study.

### Protocol

**Figure 1:**
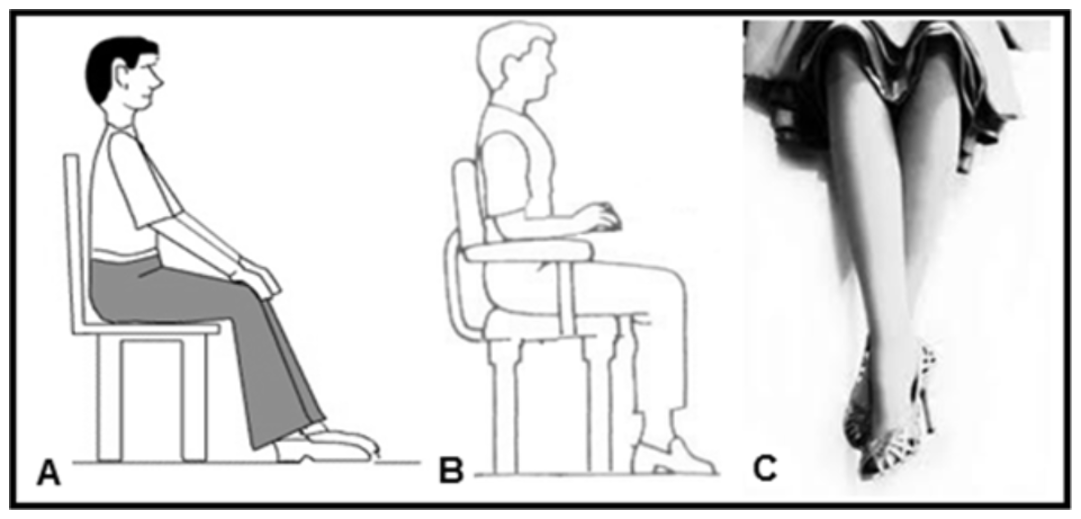
Postures used in the spirometric analyses. A: Standard posture with no upper limbs support in the arm chair and lower limbs in neutral position; B: upper limbs resting on the arm chair; and C: crossed legs.

Initially, all subjects underwent a FVC assessment in order to determine the absence of spirometric abnormalities. Anthropometric data and health history were also recorded. This initial FVC assessment was performed in the standard posture (Figure 1A) with the subject seated, no UL support and LL in neutral position, and these results were then used as the basis for all subsequent comparative analyses of the protocol.

For all assessments the Spirobank G spirometer (Medical International Research, Rome, Italy) was used. The adopted reproducibility criteria were those from the ATS/ERS guidelines [2]: for test acceptance, the two largest FEV_1_ and FVC values should differ less than 0.15 L; PEF less than 0.5 L; vital capacity (VC) less than 0,15 L, and MVV less than 20%. There is no mention of acceptable reproducibility criteria for the FEF_25–75%_ in the ATS/ERS guidelines.

The ATS/ERS guidelines recommend that at least 3 acceptable FVC and slow VC maneuvers should be performed, with 2 of them fitting the reproducibility criteria; for MVV, at least 2 acceptable and reproducible maneuvers should be obtained [2]. The ATS/ERS recommendations also bring the maximum amount of maneuvers to be performed, indicating up to 8 FVC attempts and up to 4 slow VC attempts according to individual difficulty to perform acceptable and reproducible maneuvers. There is no mention about maximum attempts for MVV [2]. The reference values adopted for spirometric results in the present study were those specific for the Brazilian population as established by Pereira et al. [8].

The study protocol consisted of four spirometric comparative analyses. In all of them, the spirometry test in the standard posture (Figure 1A) was used as a point of comparison with all intra-individual trials randomized in their sequence, i.e. randomized within each analysis and randomized between the four analyses. The interval established among the maneuvers was 30 seconds. The protocol analyses were as follows:

1. Nose clip use during MVV: In the standard posture and with the evaluator holding the mouthpiece, MVV was compared with noseclip and without it. Furthermore, the subjective discomfort caused by the use of the noseclip was assessed based on the scale described in the study by Agarwal et al. [7].
2. Upper limbs positioning: subject with noseclip and evaluator holding the mouthpiece, FVC was compared in two variations of the UL positioning: standard posture with UL relaxed along the trunk *versus* similar posture but with the UL resting on the arms of the chair (Figure 1B).
3. Lower limbs positioning: subject with noseclip and evaluator holding the mouthpiece, FVC was compared in two variations of the LL positioning: standard posture with LL in a neutral position (not crossed) *versus* similar posture but with the extended right lower limb (RLL) crossed over left lower limb (LLL) in extension (Figure 1C).
4. Who holds the mouthpiece: subject in the standard posture and with a noseclip, slow VC, FVC and MVV maneuvers were performed with the evaluated subject holding the mouthpiece with one hand *versus* the evaluator holding the mouthpiece.

The four spirometric analyses were performed within 1 day of assessment, with total duration ranging from 1 hour to 1 hour and 15 minutes.

### Sample size calculation

With the software PS Power and Sample Size Calculation, the sample size was determined based on the study of Costa et al. [3] which found differences in FEV_1_/FVC when comparing cervical extension with the cervical in neutral position in healthy individuals performing spirometric testing. The variable used for calculation was the FEV_1_/FVC with range of 1.72 in the comparison of postures, standard deviation of 4.8, p = 0.05 and power of 0.80, resulting in 63 participants as the necessary sample. The study by Costa et al. [3] was chosen since it had some similarities with the present study, e.g., same evaluated spirometric variables and sample composed by healthy individuals. Other available studies in the literature differed notably in the studied population, and it should be highlighted that no study so far had the same objective proposed here, what made the sample size calculation particularly difficult to perform. For these reasons, we chose to increase the proposed final sample size to at least above 100 subjects.

### Statistical analysis

Statistical analysis was performed with the software GraphPad Prism 5.0. Normality in data distribution was verified using the Shapiro-Wilk test. For all comparative analyses the paired Student t test or Wilcoxon test was used according to the results of the normality test. The statistical significance adopted was p<0.05.

The randomization of the maneuvers was done using the Microsoft Office Excel 2007 software, generating a randomized sequence for the 4 analyses (1, 2, 3 and 4) as well as a randomized sequence for the intra analysis (A and B, e.g., A: with noseclip, B: without noseclip).

## Results

A hundred and nine individuals were included. Six subjects were excluded: 5 had abnormal spirometry results in the initial assessment (2 with airway obstruction and 3 with restriction) and 1 for quitting the protocol due to difficulties in the proposed tests. Details about the sample characteristics are shown in Table 1.

**Table 1:** Sample characteristics (n = 103).

Variables	
Gender (men/women)	41/62
Age (years)	47 (33–58)
Weight (Kg)	73 (62–83)
Height (m)	1.66±0.1
BMI (Kg/m^2^)	26 (23–30)
FEV_1_/FVC (%)	83±5
FEV_1_ (% predicted)	94 (88–104)
FVC (% predicted)	92 (84–102)

Data shown as median (interquartile range 25%–75%), or mean±standard deviation. BMI: body mass index. FEV_1_: forced expiratory volume in the first second. FVC: forced vital capacity.

### Analysis 1: noseclip use during MVV and evaluation of the discomfort caused by the noseclip

Comparing MVV with and without the use of a noseclip, no statistically significant differences were found. When the sample was separated into age groups (18–29 years, 30–39, 40–49, 50–59, 60–72), there was a borderline difference (p = 0.05) only in the age group of 18–29 years, however with a difference that did not exceed the ATS/ERS acceptable values of reproducibility (Table S16 in File S1).

Table 2 shows the results of subjective reports on the discomfort caused by the noseclip use during the MVV maneuver. Most participants (61.2%) did not report any discomfort caused by the noseclip, whereas only 19.4% of them reported that they preferred to perform the test without the noseclip.

**Table 2:** Discomfort caused by the noseclip use in the MVV maneuver.

Score	Frequency (%)
0 no discomfort	61.2
1 unwell sensation	19.4
2 discomfort present, better without noseclip	19.4
3 discomfort present, will do only after persuasion	0
4 can not perform due to discomfort	0

MVV: maximum voluntary ventilation.

### Analysis 2 and 3: upper and lower limbs positioning

Some variables showed statistically significant differences when comparing UL with and without support (Table 3) and when comparing LL positioning (Table 4). However, these differences did not exceed the acceptable values of reproducibility.

**Table 3:** Comparison between forced vital capacity performed without (standard) or with upper limbs support.

Variables	Without support	With support	Δ (without-with)	p
FVC(L)	3.46(3–4.48)	3.43(3–4.4)	0.02	0.012
FEV_1_ (L)	2.89(2.54–3.64)	2.89(2.5–3.54)	0.05	<0.0001
FEV_1_/FVC(%)	83(79–86)	83(79.5–86)	0.5	0.5
PEF(L/s)	6.79(5.65–8)	6.63(5.59–8.26)	0.2	0.03
FEF_25–75%_ (L/s)	3.26(2.93–3.75)	3.25(2.67–3.74)	0.04	0.2

Data shown as median (interquartile range 25%–75%). FVC: forced vital capacity. FEV_1_: forced expiratory volume in the first second. PEF: peak expiratory flow. FEF_25–75%_: forced expiratory flow between 25% and 75% of FVC.

**Table 4:** Comparison between forced vital capacity performed with lower limbs in neutral position (standard) or crossed.

Variables	Neutral position	Crossed	Δ(neutral-cross)	p
FVC(L)	3.46(3–4.48)	3.44(3–4.39)	0.04	0.012
FEV_1_ (L)	2.89(2.54–3.64)	2.87(2.46–3.48)	0.05	<0.0001
FEV_1_/FVC(%)	83(79–86)	83(80–86)	0.1	0.38
PEF(L/s)	6.79(5.65–8)	6.62(5.58–8.63)	0.08	0.4
FEF_25–75%_ (L/s)	3.26(2.93–3.75)	3.18(2.58–3.89)	0.06	0.03

Data shown as median (interquartile range 25%–75%). FVC: forced vital capacity. FEV_1_: forced expiratory volume in the first second. PEF: peak expiratory flow. FEF_25–75%_: forced expiratory flow between 25% and 75% of FVC. cross: lower limbs crossed.

Furthermore, when analyzing the various age groups, the observed statistically significant differences also did not exceed the acceptable values of reproducibility [2]. Results of the various age groups for UL positioning (Tables S2, S5, S8, S11 and S14 in File S1) and LL positioning (Tables S3, S6, S9, S12 and S15 in File S1) can be found in the supporting information.

### Analysis 4: evaluator holding the mouthpiece compared to evaluated subject holding it

Both in the whole sample (Table 5) and in the age groups (Tables S1, S4, S7, S10, S13 in File S1), some variables had statistically significant differences when comparing the evaluator or subject holding the mouthpiece. However, none of these differences exceeded the acceptable values of reproducibility.

**Table 5:** Comparison between forced vital capacity performed with the evaluator holding the mouthpiece and evaluated subject holding it.

Variables	Evaluator holding	Subject holding	Δ (evaluator-subject)	p
FVC(L)	3.46(3–4.48)	3.47(2.92–4.31)	0.03	0.056
FEV_1_ (L)	2.89(2.54–3.64)	2.89(2.41–3.52)	0.03	0.003
FEV_1_/FVC(%)	83(79–86)	83(80–87)	0	0.63
PEF(L/s)	6.79(5.65–8)	6.62(5.66–8)	−0.01	0.94
FEF_25–75%_ (L/s)	3.26(2.93–3.75)	3.18(2.75–4.1)	−0.02	0.84

Data as median (interquartile range 25%–75%). FVC: forced vital capacity. FEV_1_: forced expiratory volume in the first second. PEF: peak expiratory flow. FEF_25–75%_: forced expiratory flow between 25% and 75% of FVC.

In the slow VC maneuver, the VC showed no statistically significant difference in the whole group. Significant differences were found only in the age groups of 18–29 years and 30–39 years (p = 0.02 and p = 0.03, respectively; Table S18 in File S1). However, once again these differences did not exceed the acceptable values of reproducibility.

No statistically significant difference was found neither in the whole sample nor in the age groups in the MVV maneuver (Table S17 in File S1).

## Discussion

In this study, the use of a noseclip in the MVV maneuver, different positioning of UL and LL during the FVC maneuver and the comparison between evaluator or evaluated subject holding the mouthpiece presented some statistical significant differences; however, these differences were within the acceptable values recommended by the ATS/ERS guidelines [2] and therefore had no relevance to interfere with the spirometric diagnosis. These results involved different spirometric variables both in the whole group and in various age groups, presenting no systematic pattern but a random behavior. Among all spirometric variables studied here, only the FEF_25–75%_ does not have reproducibility criteria recommended by the ATS/ERS guidelines [2], and therefore applying these conclusions directly for this variable is not possible. However, the FEF_25–75%_ variation in all comparative analyses was very small (Δ = 20 ml to 60 ml) and probably also clinically irrelevant. Therefore, small variations in the spirometry technique can be tolerated in healthy adults, as they do not interfere significantly with the spirometric results.

**Figure 2:**
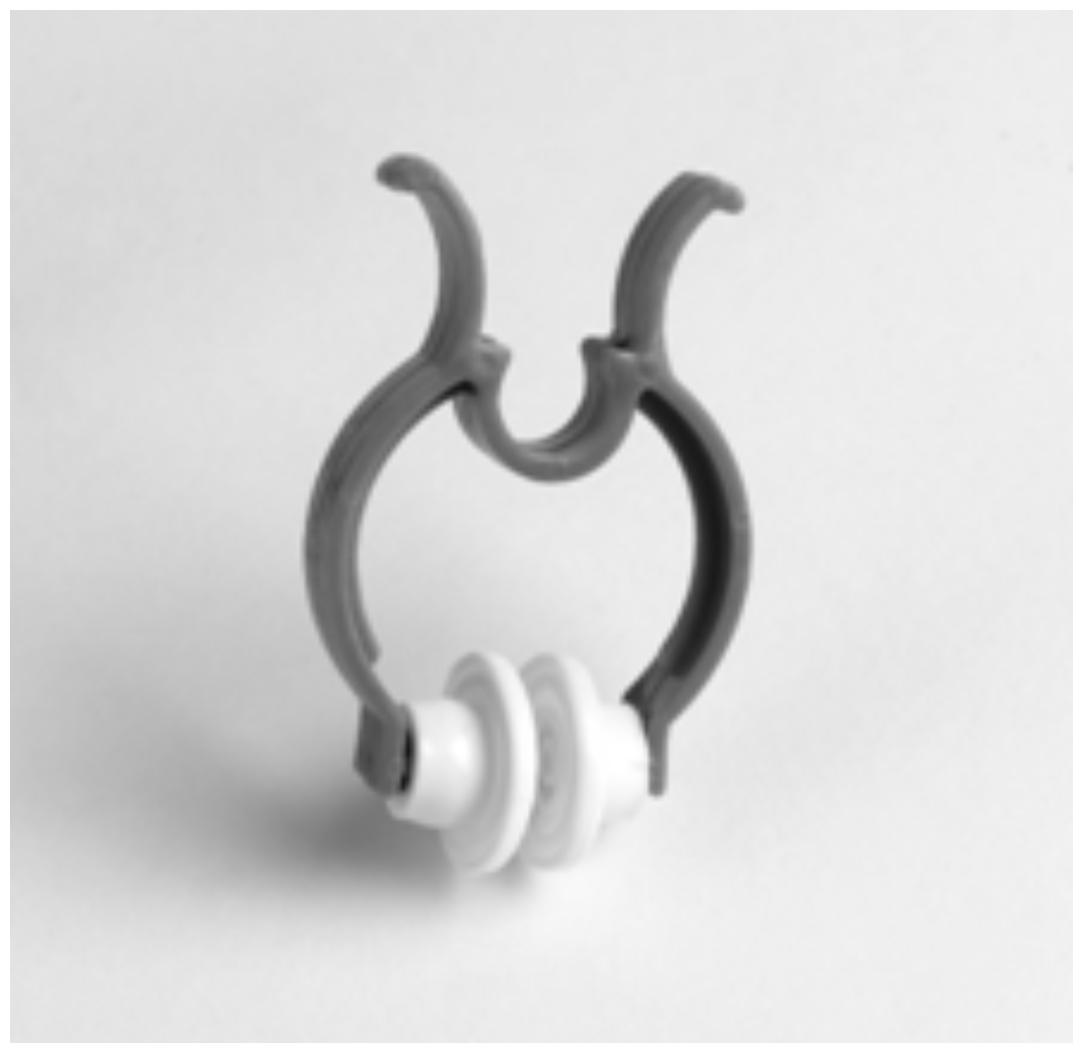
Model of noseclip used in the present study.

Regarding the noseclip use in the MVV maneuver, the only study found in the literature with a similar sample was the one by Agarwal et al. (healthy subjects aged 30±7 years) [7]. Their results corroborate the present study since no significant differences were found in MVV results performed with and without the noseclip. As for the scale of subjective discomfort described in their study [7], they found that 97% of subjects reported some kind of discomfort with the clip, which differs considerably from the results found in our sample with the same subjective scale (38.8% reported some kind of discomfort, with only half of these reporting to prefer performing the test without the noseclip). Hypothetically, this variation between studies may be due to the noseclip material, which can cause more or less discomfort according to its stiffness or softness. The contact area of the noseclip used in this study was soft, though it sealed perfectly the nostrils (Figure 2). However, this cannot be confirmed since we do not have information on the noseclip model used in the study by Agarwal et al. [7], despite trying to contact the authors. On the other hand, this contrasting result cannot be due to differences in age characteristics between the two studies, since even when comparing the discomfort reported by a specific similar age range in both samples (18–39 years) we also found that the proportion of subjects reporting discomfort was markedly lower (42% vs. 97%) in the present study.

About UL positioning, Cavalheri et al. evaluated patients with COPD performing spirometry (slow VC, FVC and MVV maneuvers) with and without upper limb support [9]. The support was done in a walker device attached to the ground, with the handle height adjusted in the line of the ulnar styloid process, with trunk inclination and elbows flexion, promoting weight bearing in the UL. However, these authors evaluated patients in the standing posture, differently from the present study which evaluated healthy subjects in the sitting position, recommended by the ATS as the preferential position [2]. Their results showed significant increase in MVV in these patients with UL support, and the authors attributed this to the fact that MVV is also a reflection of the assessment of respiratory muscle weakness [9]. However, as observed in our study, the difference between with and without arm support in COPD patients was below 20%, which once again fits the ATS/ERS reproducibility criteria, with no clinically relevant interference in the spirometric results [2].

As for the LL positioning, no study was found in the literature with similar objective and ours is the first to touch upon this topic. Based on our results, no strict control of the LL positioning is necessary and the subject may take the test in the most comfortable position since this does not interfere relevantly with the results.

Regarding the analysis about who holds the mouthpiece during FVC, slow VC and MVV maneuvers, evaluator or subject, once again we verified that the literature does not provide any information on this topic. Panka et al. studied changes in breathing pattern in healthy subjects (mean age 60 years, ranging from 51 to 71) when the activity of combing hair with unsupported UL elevation was compared to the resting condition [10]. Results showed significant increase in tidal volume, minute ventilation, respiratory rate and mean inspiratory flow after the first minute of combing hair with unsupported UL elevation [10]. The activity evaluated by these authors differs considerably from the analysis verified in the present study (i.e., holding the mouthpiece during spirometry testing); however it provides information on UL elevation in healthy individuals and its interference with the breathing pattern. Changes in the breathing pattern appeared only after the first minute of the functional activity, increasing after the third minute [10]. Considering that spirometric maneuvers last considerably less than a minute each, this duration could not be long enough in healthy individuals to allow the UL effort of holding the mouthpiece to interfere in the results as shown by Panka et al. [10].

### Limitations

The difficulty in standardizing the interval between tests can be seen as a limitation. At first, 30 seconds of rest were determined between each maneuver; however we noticed that in some subjects this was not possible as they presented fatigue, were thirsty or coughing, so this interval was slightly larger in some cases. However, we believe that this issue did not affect the results since the tests were randomized both intra-test and between the four analyses. In addition, some borderline statistical results could be due to the sample size calculated based on a study with limited similarity, since no studies were found with more similarities. However, due to the very small and acceptable differences observed when analyzing the 4 types of comparisons, we believe that an increase in sample size would not yield any clinically relevant change in the spirometric results. Finally, our study refers to a sample of individuals with normal spirometric values. Future research involving patients with respiratory disorders should be encouraged to better clarify the impact of technical details on spirometry results in these populations, contributing to the standardization of the test in different groups of patients.

## Conclusions

Technical details of spirometry testing such as the noseclip use in the MVV maneuver, upper and lower limbs positioning during the FVC maneuver and if the evaluator or the evaluated subject should hold the mouthpiece did not interfere relevantly in the spirometric results in healthy adults. Although some statistically significant differences were found, these differences did not exceed the acceptable reproducibility criteria and therefore were not clinically relevant. In summary, the decision about the way to perform the spirometry test in these individuals can be taken by the evaluator and the evaluated subject without interfering in the test results.

## Supporting Information

File S1 **Supporting tables. Table S1, Comparison between forced vital capacity performed with evaluator holding the mouthpiece and subject holding it for age group of 18–29 years (n = 19). Table S2, Comparison between forced vital capacity performed without (standard) or with upper limbs support for age group of 18–29 years (n = 19). Table S3, Comparison between forced vital capacity performed with lower limbs in neutral position (standard) or crossed for age group of 18–29 years (n = 19). Table S4, Comparison between forced vital capacity performed with evaluator holding the mouthpiece and subject holding it for age group of 30–39 years (n = 18). Table S5, Comparison between forced vital capacity performed without (standard) or with upper limbs support for age group of 30–39 years (n = 18). Table S6, Comparison between forced vital capacity performed with lower limbs in neutral position (standard) or crossed for age group of 30–39 years (n = 18). Table S7, Comparison between forced vital capacity performed with evaluator holding the mouthpiece and subject holding it for age group of 40–49 years (n = 23). Table S8, Comparison between forced vital capacity performed without (standard) or with upper limbs support for age group of 40–49 years (n = 23). Table S9, Comparison between forced vital capacity performed with lower limbs in neutral position (standard) or crossed for age group of 40–49 years (n = 23). Table S10, Comparison between forced vital capacity performed with evaluator holding the mouthpiece and subject holding it for age groups of 50–59 years (n = 20). Table S11, Comparison between forced vital capacity performed without (standard) or with upper limbs support for age group of 50–59 years (n = 20). Table S12, Comparison between forced vital capacity performed with lower limbs in neutral position (standard) or crossed for age group of 50–59 years (n = 20). Table S13, Comparison between forced vital capacity performed with evaluator holding the mouthpiece and subject holding it for age group of 60–72 years (n = 23). Table S14, Comparison between forced vital capacity performed without (standard) or with upper limbs support for age group of 60–72 years (n = 23). Table S15, Comparison between forced vital capacity performed with lower limbs in neutral position (standard) or crossed for age group of 60–72 years (n = 23). Table S16, Comparison between maximum voluntary ventilation, in liters/minute, with and without noseclip according to age groups. Table S17, Comparison between maximum voluntary ventilation, in liters/minute, performed with evaluator holding the mouthpiece and subject holding it according to age groups. Table S18, Comparison of variable VC, in liters, of the maneuver slow vital capacity, performed with evaluator holding the mouthpiece and subject holding it according to age groups.**
(DOC)
