# Supplementary material for: Spirometry in Healthy Subjects: Do Technical Details of the Test Procedure Affect the Results?
Source: PLoS One. 2014 Sep 22;9(9):e107782. doi: 10.1371/journal.pone.0107782 (PMC4171496; doi:10.1371/journal.pone.0107782)
Supplement: File S1 — Supporting tables. Table S1, Comparison between forced vital capacity performed with evaluator holding the mouthpiece and subject holding it for age group of 18–29 years (n = 19). Table S2, Comparison between forced vital capacity performed without (standard) or with upper limbs support for age group of 18–29 years (n = 19). Table S3, Comparison between forced vital capacity performed with lower limbs in neutral position (standard) or crossed for age group of 18–29 years (n = 19). Table S4, Comparison between forced vital capacity performed with evaluator holding the mouthpiece and subject holding it for age group of 30–39 years (n = 18). Table S5, Comparison between forced vital capacity performed without (standard) or with upper limbs support for age group of 30–39 years (n = 18). Table S6, Comparison between forced vital capacity performed with lower limbs in neutral position (standard) or crossed for age group of 30–39 years (n = 18). Table S7, Comparison between forced vital capacity performed with evaluator holding the mouthpiece and subject holding it for age group of 40–49 years (n = 23). Table S8, Comparison between forced vital capacity performed without (standard) or with upper limbs support for age group of 40–49 years (n = 23). Table S9, Comparison between forced vital capacity performed with lower limbs in neutral position (standard) or crossed for age group of 40–49 years (n = 23). Table S10, Comparison between forced vital capacity performed with evaluator holding the mouthpiece and subject holding it for age groups of 50–59 years (n = 20). Table S11, Comparison between forced vital capacity performed without (standard) or with upper limbs support for age group of 50–59 years (n = 20). Table S12, Comparison between forced vital capacity performed with lower limbs in neutral position (standard) or crossed for age group of 50–59 years (n = 20). Table S13, Comparison between forced vital capacity performed with evaluator holding the mouthpiece and subje [file pone.0107782.s001.doc]

**SUPPORTING INFORMATION**

**RESULTS BY AGE GROUP**

**Age group of 18-29 years**

Table S1. Comparison between forced vital capacity performed with evaluator holding the mouthpiece and subject holding it for age group of 18-29 years (n=19).

Variables

FVC(L)

FEV_1_ (L)

FEV_1_/FVC (%)

PEF(L/s)

FEF_25-75%_ (L/s)

Data as median (interquartile range 25% -75%). FVC: forced vital capacity. FEV_1_: forced expiratory volume in one second. PEF: peak expiratory flow. FEF_25-75%_: forced expiratory flow between 25% and 75% of FVC. n: sample size.

Evaluator

holding

3.68(3.34-5.24)

3.21(2.85-4.48)

85(83-87)

7.39(6.49-10.2)

3.42(3.1-4.4)

Subject

holding

3.62(3.37-5.47)

3.15(2.92-4.37)

84(83-88)

7.22(6.26-10.4)

3.48(2.94-4.66)

∆ (evaluator- subject)

0.01

0.07

0.5

0.27

0.02

p

0.5

0.13

0.2

0.09

0.6

Table S2. Comparison between forced vital capacity performed without (standard) or with upper limbs support for age group of 18-29 years (n=19).

Variables

FVC(L)

FEV_1_ (L)

FEV_1_/FVC (%)

PEF(L/s)

FEF_25-75%_ (L/s)

Data as median (interquartile range 25% -75%), or mean±standard deviation. FVC: forced vital capacity. FEV_1_: forced expiratory volume in one second. PEF: peak expiratory flow. FEF_25-75%_: forced expiratory flow between 25% and 75% of FVC. n: sample size.

Without support

3.68(3.34-5.24)

3.21(2.85-4.48)

85(83-87)

8.37±2.43

3.42(3.1-4.4)

With support

3.55(3.3-5.29)

3.26(2.9-4.33)

85(83-87)

7.88±2.33

3.58(3-4.3)

∆ (without-with)

0.06

0.07

0.24

0.49

0.07

p

0.04

0.03

0.81

0.0004

0.24

Table S3. Comparison between forced vital capacity performed with lower limbs in neutral position (standard) or crossed for age group of 18-29 years (n=19).

Variables

FVC(L)

FEV_1_ (L)

FEV_1_/FVC (%)

PEF(L/s)

FEF_25-75%_ (L/s)

Data as median (interquartile range 25% -75%), or mean±standard deviation. FVC: forced vital capacity. FEV_1_: forced expiratory volume in one second. PEF: peak expiratory flow. FEF_25-75%_: forced expiratory flow between 25% and 75% of FVC. cross: lower limbs crossed. n: sample size.

Neutral position

3.68(3.34-5.24)

3.21(2.85-4.48)

85(83-87)

8.37±2.43

3.42(3.1-4.4)

Crossed

3.59(3.21-5.44)

3.12(2.87-4.49)

86(83-88)

8.1±2.33

3.46(2.97-4.56)

∆ (neutral-cross)

0.05

0

0.21

0.26

0.01

p

0.21

0.23

0.97

0.03

0.74

**Age group of 30-39 years**

Table S4. Comparison between forced vital capacity performed with evaluator holding the mouthpiece and subject holding it for age group of 30-39 years (n=18).

Variables

FVC(L)

FEV_1_ (L)

FEV_1_/FVC (%)

PEF(L/s)

FEF_25-75%_ (L/s)

Data as mean±standard deviation. FVC: forced vital capacity. FEV_1_: forced expiratory volume in one second. PEF: peak expiratory flow. FEF_25-75%_: forced expiratory flow between 25% and 75% of FVC. n: sample size.

Evaluator

holding

4.4±1.08

3.59±0.82

82±6

6.99±1.5

3.85±0.93

Subject

holding

4.32±1.07

3.57±0.83

83±7

7.26±1.56

4.04±1.2

∆ (evaluator-subject)

0.06

0.02

-1.05

-0.27

-0.03

p

0.07

0.44

0.09

0.17

0.31

Data as median (interquartile range 25% -75%). FVC: forced vital capacity. L: liters. s: seconds. FEV_1_: forced expiratory volume in one second. PEF: peak expiratory flow. FEF_25-75%_: forced expiratory flow between 25% and 75% of FVC. n: sample size.

Table S5. Comparison between forced vital capacity performed without (standard) or with upper limbs support for age group of 30-39 years (n=18).

Variables

FVC(L)

FEV_1_ (L)

FEV_1_/FVC (%)

PEF(L/s)

FEF_25-75%_ (L/s)

Data as median (interquartile range 25% -75%), or mean±standard deviation. FVC: forced vital capacity. FEV_1_: forced expiratory volume in one second. PEF: peak expiratory flow. FEF_25-75%_: forced expiratory flow between 25% and 75% of FVC. n: sample size.

Without support

4.4±1.08

3.59±0.82

83(78-85)

6.99±1.5

3.85±0.93

With support

4.29±1.03

3.53±0.79

84(74-87)

7.25±1.45

3.93±1.09

∆(without-with)

0.11

0.06

-1.3

-0.26

0.0005

p

0.07

0.2

0.46

0.2

0.56

Table S6. Comparison between forced vital capacity performed with lower limbs in neutral position (standard) or crossed for age group of 30-39 years (n=18).

Variables

FVC(L)

FEV_1_ (L)

FEV_1_/FVC (%)

PEF(L/s)

FEF_25-75%_ (L/s)

Data as mean±standard deviation. FVC: forced vital capacity. FEV_1_: forced expiratory volume in one second. PEF: peak expiratory flow. FEF_25-75%_: forced expiratory flow between 25% and 75% of FVC. cross: lower limbs crossed. n: sample size.

Neutral position

4.4±1.08

3.59±0.82

82±6

6.99±1.5

3.85±0.93

Crossed

4.3±1.06

3.53±0.81

83±6

7.36±2.09

3.9±1.07

∆ (neutral-cross)

0.1

0.05

-0.7

-0.04

-0.01

p

0.06

0.10

0.29

0.35

0.7

**Age group of 40-49 years**

Table S7. Comparison between forced vital capacity performed with evaluator holding the mouthpiece and subject holding it for age group of 40-49 years (n=23).

Variables

FVC(L)

FEV_1_ (L)

FEV_1_/FVC (%)

PEF(L/s)

FEF_25-75%_ (L/s)

Data as median (interquartile range 25% -75%), or mean±standard deviation. FVC: forced vital capacity. FEV_1_: forced expiratory volume in one second. PEF: peak expiratory flow. FEF_25-75%_: forced expiratory flow between 25% and 75% of FVC. n: sample size.

Evaluator

holding

3.71±0.76

2.95(2.64-3.33)

84±5

7.12±1.58

3.29(3.14-3.99)

Subject

holding

3.69±0.75

2.89(2.6-3.31)

83±5

7.16±1.83

3.18(2.84-3.88)

∆ (evaluator- subject)

0.06

0.04

-0.5

-0.04

0.04

p

0.5

0.03

0.32

0.84

0.84

Table S8. Comparison between forced vital capacity performed without (standard) or with upper limbs support for age group of 40-49 years (n=23).

Variables

FVC(L)

FEV_1_ (L)

FEV_1_/FVC (%)

PEF(L/s)

FEF_25-75%_ (L/s)

Without support

3.71±0.76

2.95(2.64-3.33)

84±5

7.12±1.58

3.29(3.14-3.99)

With support

3.7±0.76

2.85(2.62-3.36)

83±5

6.92±1.83

3.11(2.99-3.65)

∆ (without-with)

0.01

0.03

0.7

0.2

0.09

p

0.46

0.06

0.17

0.37

0.2

Data as median (interquartile range 25% -75%), or mean±standard deviation. FVC: forced vital capacity. FEV_1_: forced expiratory volume in one second. PEF: peak expiratory flow. FEF_25-75%_: forced expiratory flow between 25% and 75% of FVC. n: sample size.

Table S9. Comparison between forced vital capacity performed with lower limbs in neutral position (standard) or crossed for age group of 40-49 years (n=23).

Variables

FVC(L)

FEV_1_ (L)

FEV_1_/FVC (%)

PEF(L/s)

FEF_25-75%_ (L/s)

Data as median (interquartile range 25% -75%), or mean±standard deviation. FVC: forced vital capacity. FEV_1_: forced expiratory volume in one second. PEF: peak expiratory flow. FEF_25-75%_: forced expiratory flow between 25% and 75% of FVC. cross: lower limbs crossed. n: sample size.

Neutral position

3.71±0.76

2.95(2.64-3.33)

84±5

7.12±1.58

3.47±0.72

Crossed

3.67±0.76

2.91(2.64-3.29)

83±5

7.09±1.63

3.35±0.78

∆ (neutral-cross)

0.04

0.06

0.1

0.03

0.12

p

0.2

0.01

0.08

0.85

0.05

**Age group of 50-59 years**

Table S10. Comparison between forced vital capacity performed with evaluator holding the mouthpiece and subject holding it for age groups of 50-59 years (n=20).

Variables

FVC(L)

FEV_1_ (L)

FEV_1_/FVC (%)

PEF(L/s)

FEF_25-75%_ (L/s)

Data as median (interquartile range 25% -75%), or mean±standard deviation. FVC: forced vital capacity. FEV_1_: forced expiratory volume in one second. PEF: peak expiratory flow. FEF_25-75%_: forced expiratory flow between 25% and 75% of FVC. n: sample size.

Evaluator

holding

3.15±0.57

2.63±0.41

84±5

5.22(4.74-6.69)

3±0.52

Subject

holding

3.17±0.56

2.62±0.42

83±3

6.13(5.34-7.35)

2.9±0.51

∆ (evaluator- subject)

-0.02

0.01

1

-0.49

0.1

p

0.67

0.67

0.3

0.11

0.14

Table S11. Comparison between forced vital capacity performed without (standard) or with upper limbs support for age group of 50-59 years (n=20).

Variables

FVC(L)

FEV_1_ (L)

FEV_1_/FVC (%)

PEF(L/s)

FEF_25-75%_ (L/s)

Without support

3.15±0.57

2.63±0.41

84±5

5.22(4.74-6.69)

3±0.52

With support

3.15±0.55

2.61±0.42

83±3

5.78(5.15-6.87)

2.94±0.52

∆ (without-with)

0.001

0.02

0.45

-0.2

0.06

p

0.98

0.43

0.37

0.56

0.31

Data as median (interquartile range 25% -75%), or mean±standard deviation. FVC: forced vital capacity. FEV_1_: forced expiratory volume in one second. PEF: peak expiratory flow. FEF_25-75%_: forced expiratory flow between 25% and 75% of FVC. n: sample size.

Table S12. Comparison between forced vital capacity performed with lower limbs in neutral position (standard) or crossed for age group of 50-59 years (n=20).

Variables

FVC(L)

FEV_1_ (L)

FEV_1_/FVC (%)

PEF(L/s)

FEF_25-75%_ (L/s)

Neutral position

3.15±0.57

2.63±0.41

84±5

5.22(4.74-6.69)

3±0.52

Crossed

3.16±0.55

2.61±0.4

83±3

5.91(4.92-7.42)

2.92±0.47

∆ (neutral-cross)

-0.002

0.02

0.82

-0.16

0.08

p

0.97

0.5

0.32

0.27

0.32

Data as median (interquartile range 25% -75%), or mean±standard deviation. FVC: forced vital capacity. FEV_1_: forced expiratory volume in one second. PEF: peak expiratory flow. FEF_25-75%_: forced expiratory flow between 25% and 75% of FVC. cross: lower limbs crossed. n: sample size.

**Age group of 60-72 years**

Table S13. Comparison between forced vital capacity performed with evaluator holding the mouthpiece and subject holding it for age group of 60-72 years (n=23).

Variables

FVC(L)

FEV_1_ (L)

FEV_1_/FVC (%)

PEF(L/s)

FEF_25-75%_ (L/s)

Data as median (interquartile range 25% -75%), or mean±standard deviation. FVC: forced vital capacity. FEV_1_: forced expiratory volume in one second. PEF: peak expiratory flow. FEF_25-75%_: forced expiratory flow between 25% and 75% of FVC. n: sample size.

Evaluator

holding

3.06(2.57-3.76)

2.61(2.21-3.58)

81±6

6.98±1.88

2.89±0.82

Subject

holding

2.9(2.56-4.02)

2.37(2.16-3.36)

81±5

6.57±1.75

2.93±0.89

∆ (evaluator- subject)

0

0.06

-0.27

0.41

-0.05

p

0.6

0.12

0.72

0.04

0.67

Table S14. Comparison between forced vital capacity performed without (standard) or with upper limbs support for age group of 60-72 years (n=23).

Variables

FVC(L)

FEV_1_ (L)

FEV_1_/FVC (%)

PEF(L/s)

FEF_25-75%_ (L/s)

Without support

3.06(2.57-3.76)

2.61(2.21-3.58)

81±6

6.98±1.88

2.89±0.82

With support

2.98(2.55-3.93)

2.45(2.2-3.28)

81±5

6.58±1.91

2.86±0.87

∆ (without-with)

0

0.08

0.25

0.4

0.02

p

0.24

0.009

0.72

0.05

0.83

Data as median (interquartile range 25% -75%), or mean±standard deviation. FVC: forced vital capacity. FEV_1_: forced expiratory volume in one second. PEF: peak expiratory flow. FEF_25-75%_: forced expiratory flow between 25% and 75% of FVC. n: sample size.

Table S15. Comparison between forced vital capacity performed with lower limbs in neutral position (standard) or crossed for age group of 60-72 years (n=23).

Variables

FVC(L)

FEV_1_ (L)

FEV_1_/FVC (%)

PEF(L/s)

FEF_25-75%_ (L/s)

Neutral position

3.06(2.57-3.76)

2.61(2.21-3.58)

81±6

6.98±1.88

2.89±0.82

Crossed

3.09(2.51-4.37)

2.42(2.2-3.44)

81±5

6.65±1.61

2.83±0.89

∆ (neutral-cross)

0.05

0.08

0.02

0.33

0.09

p

0.45

0.006

0.97

0.1

0.52

Data as median (interquartile range 25% -75%), or mean±standard deviation. FVC: forced vital capacity. FEV_1_: forced expiratory volume in one second. PEF: peak expiratory flow. FEF_25-75%_: forced expiratory flow between 25% and 75% of FVC. cross: lower limbs crossed. n: sample size.

**Maximum voluntary ventilation with and without noseclip according to age groups**

Table S16. Comparison between maximum voluntary ventilation, in liters/minute, with and without noseclip according to age groups.

Age groups

18-29 years

30-39 years

40-49 years

50-59 years

60-72 years

Data as median (interquartile range 25% -75%), or mean±standard deviation. n: sample size. In none of the age groups, the difference between with and without noseclip exceeded 20%, which is the maximum difference accepted in the ATS/ERS reproducibility criteria.

n

19

18

23

20

23

With

145±40

137.5±34

115(94-139)

103±32

113±36

Without

138±37

135±38

116(95-146)

102±32

112±34

∆(with-without)

7.7

2.6

2

1.5

0.8

p

0.05

0.28

0.2

0.5

0.74

**Maximum voluntary ventilation with evaluator or evaluated subject holding the mouthpiece. according to age groups**

Table S17. Comparison between maximum voluntary ventilation, in liters/minute, performed with evaluator holding the mouthpiece and subject holding it according to age groups.

Age groups

18-29 years

30-39 years

40-49 years

50-59 years

60-72 years

Data as mean±standard deviation. n: sample size. In none of the age groups, the difference between evaluator and subject holding the mouthpiece exceeded 20%, which is the maximum difference accepted in the ATS/ERS reproducibility criteria.

n

19

18

23

20

23

Evaluator

holding

145±40

138±34

119±41

103±32

113±36

Subject

holding

145±40

137±36

125±36

103±26

112±37

∆ (evaluator-subject)

-0.005

1.03

-1.5

0.24

0.8

p

0.99

0.76

0.48

0.93

0.66

**Slow vital capacity with evaluator or evaluated subject holding the mouthpiece. according to age groups**

Table S18. Comparison of variable VC, in liters, of the maneuver slow vital capacity, performed with evaluator holding the mouthpiece and subject holding it according to age groups.

Age groups

18-29 years

30-39 years

40-49 years

50-59 years

60-72 years

Data as median (interquartile range 25% -75%), or mean±standard deviation. VC: vital capacity. n: sample size.

n

19

18

23

20

23

Evaluator

holding

3.79(3.13-5.76)

4.42±1.04

3.81±0.78

3.14(2.58-3.6)

3.24(2.68-4.36)

Subject

holding

3.84(3.2-5.8)

4.32±1.05

3.76±0.83

3.35(2.64-4)

3.15(2.8-4.14)

∆ (evaluator- subject)

-0.07

0.1

0.02

-0.05

-0.02

p

0.02

0.03

0.3

0.08

0.9
